# Characterization of a novel thermostable alkaline lipase derived from a compost metagenomic library and its potential application in the detergent industry

**DOI:** 10.3389/fmicb.2022.1088581

**Published:** 2022-12-23

**Authors:** Qing-Qing Li, Zi-Ran Zhu, Qing-Gang Liu, Yu-Ting An, Yi-Xiang Wang, Shu-Bin Zhang, Gang Li

**Affiliations:** ^1^School of Life Sciences, Sun Yat-sen University, Guangzhou, China; ^2^Guang Zhou Liby Enterprise Group Co., Ltd., Guangzhou, China

**Keywords:** lipase, compost, heat stable, alkali resistant, detergent additive enzyme

## Abstract

Using composted soil samples, a metagenomic library consisting of 36,000 clones was constructed. Then, a novel lipase, Lip54q, which belongs to the VIII family of lipolytic enzymes, was identified from the metagenomic library by functional screening. To explore the enzymatic properties of Lip54q, *lip54q* was heterologous expressed in *Escherichia coli* with a high expression level of recombinant protein up to 720 mg/L. The recombinant enzyme showed the highest activity (28,160 U/mg) against a C10 substrate at pH 9.0 and 47°C, and was stable at temperatures ≤50°C and pH 8.0–11.0. Of particular interest, the surfactants, Tween-20, Tween-80 and Tritonx-100, exhibited strong promoting effects on Lip54q activities regardless of whether low concentrations (0.1%) or high concentrations (10%) were used. Application studies of Lip54q using six commercial detergents indicated that the enzyme had strong tolerance and immersion resistance to all six detergents. The results of oil-stain removal experiments suggested that addition of the enzyme to various commercial detergents could significantly improve the abilities of these detergents to remove oil-stains. Furthermore, the results of a molecular docking analysis of Lip54q showed that both the C10 substrate and linoleic acid molecules could form hydrogen bond interactions with the catalytic amino acids, Ser-268, Glu-168, and Asp-192, in the catalytic center of the enzyme, and the hydrogen bond distances were shorter. The electrostatic attraction between the enzyme and the substrate formed by the hydrogen bond with a shorter distance is stronger, which is conducive to the formation of a more stable complex between the enzyme and the substrate, thus increasing the activity of the enzyme to such substrate. These results 1ay a good foundation for application of this enzyme in the detergent industry in the future.

## Introduction

Based on different substrates, lipolytic enzymes can be broadly divided into carboxylesterases (EC3.1.1.1), aryl esterases (EC3.1.1.2), and lipases (EC3.1.1.3). Among them, esterases mainly decompose esters with short carbon chains (carbon atom lengths <10) that are soluble in water, while lipases mainly decompose esters with long carbon chains (carbon atom lengths ≥10) that are insoluble in water and often play catalytic roles at the oil–water interface (Casas-Godoy et al., 2012). As members of the α/β hydrolase family, most esterases and lipases contain a typical α/β protein domain (Beisson et al., 2000), and generally have a triad catalytic center (Ser-asp/Glu-His) and pentapeptide-conserved sequence (GLy-X-Ser-X-Gly) near the active site, “Ser” (Javed et al., 2018). Arpigny et al. divided lipolytic enzymes into eight families based on their differences in amino acid sequences and characteristics of conserved sequences to predict the important structural characteristics of lipolytic enzymes, secretion mechanisms and potential relationships with other enzyme families (Arpigny and Jaeger, 1999). With the development of metagenomics and other technologies, some new bacterial and fungal esterase/lipase families have been discovered based on phylogenetic criteria and other conserved sequence motifs (Zarafeta et al., 2016; Parapouli et al., 2018; Jia et al., 2019), which means that the classification of esterase hydrolases is increasingly accurate.

As important biocatalysts, lipases can efficiently catalyze the hydrolysis and synthesis reactions of different substrates, which makes lipases widely used in food processing, dairy products, the feed industry, bioenergy, biomedicine, detergents, textiles and other fields and they have become one of the three important industrial enzymes after proteases and amylase (Martínez-Ruiz et al., 2018). One of the most important commercial applications of lipases is their use as detergent additives in commercial laundries, household laundries and dishwasher. Since most fatty food stains on clothes or tableware are triglycerides composed of long-chain fatty acids and glycerol and triglycerides are the natural substrates of lipases, the decontamination effect of lipases is mainly manifested in their ability to hydrolyze oils and generate hydrophilic fatty acids (Joseph and Ramteke, 2013). Addition of lipase to detergents not only can enhance the decontamination ability but can also decrease the use of inorganic auxiliaries and surfactants that pollute the environment, thereby reducing the environmental load caused by detergent products (Ugo et al., 2017; Al-Ghanayem and Joseph, 2020).

Due to the specific properties of the washing environment, lipases suitable for detergent addition should be alkali resistant (pH 10.0–11.0) and have good activity and stability at 30–60°C (Hemlata et al., 2016). Although many lipases obtained from various habitats have been identified, and some of them show potential for application in the detergent industry (Abol-Fotouh et al., 2021; Shoja and Minai-Tehrani, 2021; Zhao et al., 2021), in general, most existing lipases are not stable in strong alkaline environments and do not have a high tolerance to surfactants, which limits the number of lipases that are suitable for detergent addition. Therefore, it is still necessary to continue searching for lipases in various habitats that are suitable for the detergent industry with strong alkali tolerance and surfactant tolerance.

Composting is a process in which all types of organic wastes (such as crop straw) are gradually mineralized, humified and rendered harmless by various microorganisms and their secreted enzymes under proper temperature, humidity and ventilation conditions after adding appropriate nitrogen sources (such as activated sludge; Ryckeboer et al., 2003). The special ecological environments of composts cause the microbial communities in them to be very complex and diverse, and most communities consist of moderate- and high-temperature microorganisms, which can produce cellulase, ligninase, lipase, and other hydrolases. Thus, microorganisms in compost habitats provide a large gene pool for mining efficient, heat-resistant lipases with promising industrial applications.

However, more than 99% of the microorganisms present in compost are difficult to isolate and culture in the laboratory, but metagenomic technology provides an effective means to solve the above problems. To date, many lipolytic enzymes obtained from compost habitats have been identified using metagenomic techniques (kang et al., 2011; Okano et al., 2015; Lee et al., 2016). However, to our knowledge, these enzymes are all esterases that hydrolyze short carbon chain *p*-nitrophenyl ester substrates and are not effective in degrading long carbon chain fatty acids. The use of metagenomic techniques to discover lipases obtained from composting habitats with potential for use in the detergent industry remains unreported.

In this study, we collected compost samples from a compost demonstration plant and constructed a metagenomic library. Through functional screening, we obtained a lipase, Lip54q, with good thermostability, high alkali tolerance, organic solvent and surfactant tolerance from the library and initially explored the application prospects of Lip54q as a detergent additive enzyme.

## Materials and methods

### Strains, vectors, and chemical reagents

The strains used in this study included *Escherichia coli* (*E. coli*) DH5α and BL21 (DE3). The vectors included the cloning vector, pUC118, and expression vector, pET32a. Various restriction endonuclease and ligase enzymes were purchased from TaKaRa Bio (Dalian, China), and a soil DNA extraction kit, DNA gel extraction kit, and DNA purification kit were purchased from OMEGA (San Diego, U.S.A.). The other reagents used in this study were purchased from Sangon Biotech (Shanghai, China) or Sigma–Aldrich (St. Louis, MO, United States) and were of analytically pure grade.

### Construction of the metagenomic library of compost soil and screening of lipolytic enzyme-positive clones

The compost soil samples used in this study were collected from a demonstration composting plant located in Dongguan, Guangdong Province, China. Metagenomic DNA was extracted by a soil DNA extraction kit obtained from OMEGA, and the extraction steps were conducted in accordance with the product guide. The extracted DNA was partially digested with *Bam*H I and a gel extraction kit (Omega) was then used to recover 2.5–10 kb DNA fragments. The recovered DNA fragments were ligated to the pUC118 vector and then electroporated into *E. coli* DH5α. The transformed cells were plated on LB medium containing 1.0 mM isopropyl-β-D-1-thiogalactopyranoside (IPTG), 1% tributyrin, and 100 μg/ml ampicillin to screen for lipolytic enzyme-positive clones that produced transparent hydrolysis halos around colonies. All positive clones were sent to Invitrogen (Shanghai, China) for sequencing.

### DNA sequence analysis and phylogenetic tree construction of lipolytic enzyme positive clones

The ORF Finder tool^1^ was used to analyze the inserts of a lipolytic enzyme-positive clone (No. 54) and search for possible open reading frames (ORFs) of lipolytic enzymes. The lipolytic enzyme, Lip54q, and its several homologous protein sequences were compared using Clustalx 2.1 and ESPript 3.0 software, and the conserved sequences of Lip54q were analyzed. Then, a phylogenetic analysis of Lip54q between Lip54q and several typical members of lipolytic enzyme families was performed using MEGA 7.0 software and the neighbor-joining method (Kumar et al., 2016), and a phylogenetic tree was constructed to identify the genetic relationships and classification information of Lip54q.

### Heterologous expression of *lip54q* and purification of the recombinant enzyme

The putative *lip54q* gene was amplified using PCR, and the two primers used are listed as follows: *lip54q*-forward (5′-CGC GGATCC ATG CAA GTG GCA AAA CCG CAG GAT C-3′), and *lip54q*-reverse (5′-CCC AAGCTT GGG CCA TGC TTA TCT TCG AGA AAG-3′). The underlined sequences in the primers are two restriction sites (*Ba*mH I and *Hin*d III) that were used to construct expression vectors. The PCR products were digested and ligated to the expression vector, pET32a, and digested with the same two restriction enzymes. The ligation products were electroporated into *E. coli* BL21(DE3). The transformants were spread on LB plates containing tributyrin and cultured overnight at 37°C. The recombinant bacteria expressing Lip54q produced transparent hydrolysis circles around the colonies. The recombinant bacteria were inoculated into LB medium and cultured at 37°C to an OD_600_ of 0.8. IPTG with a final concentration of 0.2 mM was added to LB medium and induced at 30°C and 150 rpm for 18 h. The cells were centrifuged and washed with pure water 2 times and then resuspended in PBS buffer. The cells were lysed by ultrasonication, and the disrupted cells were centrifuged. The supernatant was a crude enzyme solution. This solution was purified by using an Ni-NTA affinity chromatography column (Qiagen, Germany) according to the product manual. The purified recombinant enzyme was stored at 4°C for further study.

### SDS–PAGE analysis of Lip54q

SDS–PAGE was used to evaluate the molecular weights and purification effects of Lip54q. Then, 20 μl of crude enzyme solution and 20 μl of purified enzyme solution were added to 4 μl of 6 × loading buffer, boiled for 5 min, and added to the concentrated gel well. Electrophoresis was performed at 80 V for 0.5 h and then at 120 V for 1 h. After electrophoresis, the gel was dyed overnight with Coomassie brilliant blue R-250. The SDS–PAGE results were observed and photographed.

### Enzymatic activity analysis of Lip54q

Ten microliters of appropriately diluted enzyme solution was added to a reaction solution containing 1 mM substrate *p*-nitrophenol butyrate and 40 mM Britton-Robinson buffer (40 mM H_3_BO_3_, 40 mM H_3_PO_4_, 40 mM acetic acid, pH 7.8) to a total volume of 400 μl. After mixing, they were reacted in a water bath at 45°C for 20 min. Three parallel experiments and one control experiment were set for each group. After the reactions finished, 200 μl of 20% SDS was added to terminate the reactions. The absorbances at 405 nm were measured with a microplate reader. One unit of enzymatic activity was defined as the amount of enzyme required to hydrolyze the substrate to produce 1 μmol of *p*-nitrophenol per minute.

### Characterization of the recombinant Lip54q

To detect the activities of Lip54q toward substrates with different carbon chain lengths, different substrates with final concentrations of 1 mM were added to the enzymatic reaction system at 45°C and pH 7.8. These substrates included *p*-nitrophenyl acetate (C2), *p*-nitrophenyl butyrate (C4), *p*-nitrophenyl caproate (C6), *p*-nitrophenyl octanoate (C8), *p*-nitrophenyl decanoate (C10), *p*-nitrophenyl laurate (C12), *p*-nitrophenyl myristate (C14), and *p*-nitrophenyl palmitate (C16). Three parallel experiments and one control experiment were performed for each group. After 20 min of reaction, 200 μl of 20% SDS was added to stop the reactions. The absorbances at 405 nm were measured with a microplate reader. The highest enzymatic activity value was defined as 100%, and the relative enzymatic activities were plotted to determine the optimal substrate for the recombinant Lip54q. To determine the influence of temperature on the activity and stability of the recombinant enzyme, the recombinant enzyme and its optimal substrate were reacted at pH 7.8 and 30–70°C, and the enzymatic activities were determined. The optimal temperature of the enzyme was determined according to the enzymatic activity levels. The enzyme was kept in a water bath at 30°C, 40°C, the optimum temperature, 50°C, and 60°C for 1, 2, 3, 4, and 5 h, respectively, and the residual enzymatic activities were determined at pH 7.8. Three parallel experiments and one control experiment were performed for each group. The enzymatic activity of the enzyme solution stored at 4°C was defined as 100%, and the relative enzymatic activities were plotted with respect to temperature to determine the thermal stability of the enzyme. To determine the effect of pH on the enzymatic activity and stability of the recombinant enzyme, the recombinant enzyme and its optimum substrate were reacted at the optimum temperature of the enzyme at pH 5.0–12.0, and the optimum pH of the enzyme was determined according to the enzymatic activity levels. The recombinant enzyme was placed at 4°C and pH 8.0, 9.0, 10.0, 11.0, and 12.0 for 1, 2, 3, 4, 5, and 6 h, respectively, and the residual enzymatic activities were determined at pH 7.8. Three parallel experiments and one control experiment were performed for each group. The initial enzymatic activity was defined as 100%, and the relative enzymatic activities were plotted with respect to pH to determine the pH stability of the enzyme. The optimal substrate of the recombinant enzyme was used to determine the kinetic parameters, *K*_m_ and *V*_max_. Ten microliters of suitably diluted enzyme solution was added to the optimal pH buffer, whose final optimal substrate concentrations were 0.0, 0.1, 0.5, 1.0, 1.5, 2.0, 2.5, 3.0, 3.5, 4.0, and 4.5 mM. The mixtures were reacted at the optimal temperature for 20 min, and 200 μl of 20% SDS was added to stop the reactions. The absorbance values were measured at 405 nm. The initial reactions rates of the enzyme were calculated. A Lineweaver-Burk double reciprocal diagram was drawn by using the substrate concentrations and the reaction rates, and the Michaelis constant, *K*_m_, and the maximum reaction speed, *V*_max_, were obtained.

### Effects of metal ions, organic solvents, ethylene diamine tetraacrtic acid, surfactants, and protease inhibitors on the enzymatic activities of Lip54q

At the optimal reaction temperature and pH of the enzyme, different final concentrations (1, 5, and 25 mM) of metal ions (Ca^2+^, Cu^2+^, Ni^2+^, Zn^2+^, Co^2+^, Mg^2+^, and Mn^2+^) were added to the enzyme reaction system to determine their effects on the enzymatic activity of Lip54q. At the optimal reaction temperature and pH of the enzyme, different final concentrations (10, 25, and 50 mM) of organic solvents [acetonitrile, methanol, ethanol, isopropanol, and dimethyl sulfoxide (DMSO)] were added to the reaction system to determine their effects on the enzymatic activity of Lip54q. At the optimal reaction temperature and pH of the enzyme, different final concentrations (10, 25, and 50 mM) of ethylene diamine tetraacrtic acid (EDTA) and different final concentrations (0.1, 1, and 10%, v/v) of the surfactants (Tween-20, Tween-80, and Triton X-100) were added to the enzymatic reaction system to determine their effects on the enzymatic activities of Lip54q. In addition, to determine whether the serine located in the active center of Lip54q is decisive for the enzymatic activity, we examined the effects of 1 and 4 mM serine protease inhibitor AEBSF [4-(2-aminoethyl) benzenesulfonyl hydrochloride] on enzymatic activities. All tests were repeated three times, and the enzymatic activity of an enzymatic reaction system without the above target reagents was defined as 100%.

### Resistance of the recombinant enzyme to organic solvents

To determine the resistance of the recombinant enzyme to organic solvents, 12 organic solvents with final concentrations of 20% (v/v) and 50% (v/v) were added to the undiluted pure enzyme solution, including DMSO, methanol, ethanol, acetone, benzene, toluene, n-hexane, cyclohexane, isooctane, n-decanol, chloroform, and n-butanol. After shaking at 30°C and 200 rpm, the residual enzymatic activities were determined at pH 7.8 after 1 h, 24 h, 48 h, 96 h, 7 days, and 12 days. The experiment was repeated three times for each group. The enzymatic activity of the pure enzyme solution without organic solvent was defined as 100%, and the relative activities of enzymes treated by each organic solvent were calculated.

### Tolerance of the recombinant enzyme to several common commercial detergents

Tide washing powder with a final concentration of 10 mg/ml, Blue Moon laundry detergent, Liby laundry detergent, Walch hand sanitizer, Axe dish soap, and Liby dish soap with a final concentration of 1% (V/V) were added to distilled water, followed by equal amounts (0.5 ml, 10 U) of recombinant enzyme solution to each solution. The pH values of the above mixtures were 8.5, 7.9, 8.1, 8.0, 8.1, and 8.1, respectively. After 1, 2, 3, 4, 6 and 24 h of treatment at room temperature, the residual activities of each mixture were determined under standard conditions. The experiment was repeated three times for each group. The enzyme activity of the pure enzyme solution without detergent was defined as 100%, and the relative activities of the enzyme treated with each detergent were calculated.

### Immersion resistance of the recombinant enzyme to several common commercial detergents

By using a standard of 10 g of detergent per kilogram of clothing and soaking in 5 L of tap water, Tide washing powder, Blue Moon laundry detergent, Liby laundry detergent, Walch hand sanitizer, Axe dish soap, and Liby dish soap were added to tap water. After mixing, 0.5 ml (10 U) of enzyme solution was added to each experimental group and treated for 10, 20, 30, 45, 60, and 120 min. Then, the residual enzymatic activities were determined under standard conditions. Each experiment was repeated three times. The enzymatic activity of the pure enzyme solution without detergent was defined as 100%, and the relative enzyme activities treated with each detergent were calculated.

### Determination of the oil-stain removal abilities of the recombinant enzyme on cotton cloth

Three pieces of white cotton cloth with the same size (4 cm × 4 cm) were soaked in cooking oil for 24 h and then removed to dry naturally. Next, they were numbered as A, B, and C and treated as follows: A. white cloth + tap water, B. white cloth + tap water + detergent, and C. white cloth + tap water + detergent + Lip54q (5 U). The detergents used in this experiment included Tide washing powder, Blue Moon Detergent, Liby laundry Detergent, Walch hand sanitizer, AXE dish soap, and Liby dish soap. The cotton cloths were washed for 30 min using the above treatment methods and then air-dried, and the residual oil stains on the three pieces of white cloth from each group were compared.

### Homology modeling of the three-dimensional structure of Lip54q

Using the tertiary structure of the protein Est-Y29 (Ngo et al., 2014) with 37.95% homology to Lip54q as the template, a three-dimensional structure of Lip54q was obtained using the SWISS-MODEL tool.^2^

### Molecular docking analysis of Lip54q and several substrates

Based on the accession number of Lip54q in the GenBank database, the amino acid sequence of Lip54q was obtained and entered into the online homology modeling server, I-TASSER),^3^ to model of the three-dimensional structure of Lip54q. The molecular docking results were processed and optimized using the Glide module in Schrodinger Maestro software. The proteins (receptors) were preprocessed, optimized and minimized using the Protein Preparation Wizard module (minimization constraints by the OPLS3e force field). Substrate structures were prepared according to the default settings for the LigPrep module. When screening in the Glide module, the prepared receptor was imported, and the potential binding sites of the protein were predicted according to the protein structure (Lip54q: *x* = 73.84, *y* = 56.10, *z* = 59.28). The box size was set to 20 Å × 20 Å × 20 Å, and the distance between grid points was 0.375 Å. Finally, molecular docking and screening were carried out using the standard precision (SP) method. By using the above method, the interaction modes of several substrate molecules (C8, C10, C12, and linoleic acid) and Lip54q were analyzed, and the results of the interactions between the substrate and amino acid residues in the protein were obtained, such as the generated hydrogen bonds, π-π interactions, hydrophobic interactions. Then, according to the docking scores of the substrates, whether the enzyme had a certain activity on the substrate can be speculated. The complexes formed by the substrate and enzyme after docking were visualized with PyMol 2.1 software to obtain the binding modes of the substrates and proteins.

### Accession number of *lip54q*

The accession number of *lip54q* (GenBank database) is KP266873.

## Results

### Screening of lipolytic enzymes from the compost metagenomic library

Using tributyrin as a substrate, the constructed compost metagenomic library was screened using a functional screening method. One lipolytic enzyme-positive clone that produced a clear hydrolysis cycle was screened from approximately 36,000 clones.

### Sequence analysis of the lipolytic enzyme-positive clone

**Figure 1:**
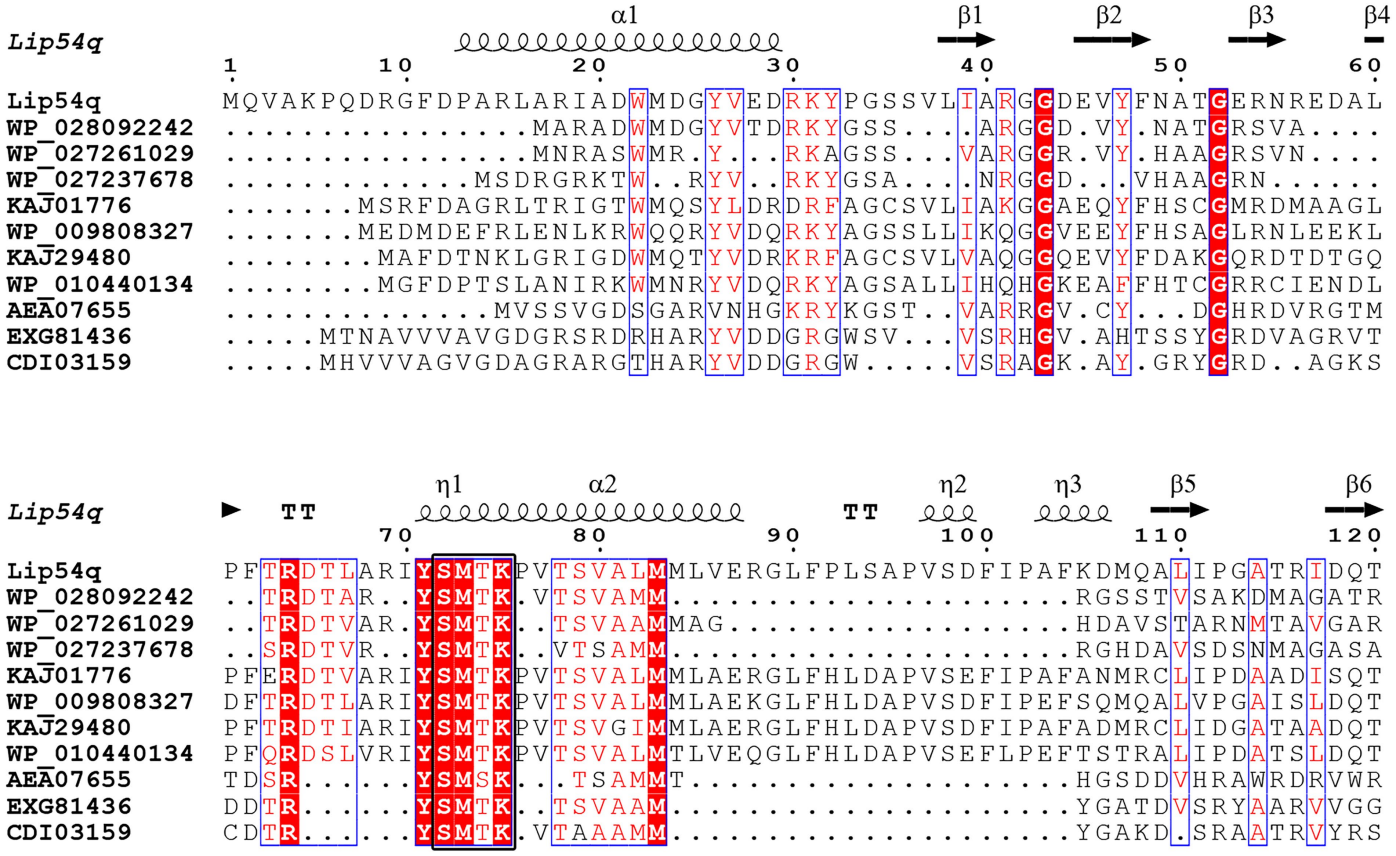
Multiple amino acid sequence alignment of Lip54q with several of its closest homologs. Several homologous amino acid sequences were obtained from NCBI GenBank. Sequence alignment was performed using Clustalx 2.1 and visualized using ESPript 3.0. The alpha helix, beta sheet, random coil and beta turn are indicated by α, β, η and T, respectively. The conserved sequences are indicated by blue boxes, and similar sequences are indicated by red backgrounds. The characteristic conserved S-M-T-K sequence of the Family VIII of lipolytic enzymes is indicated by a black box.

**Figure 2:**
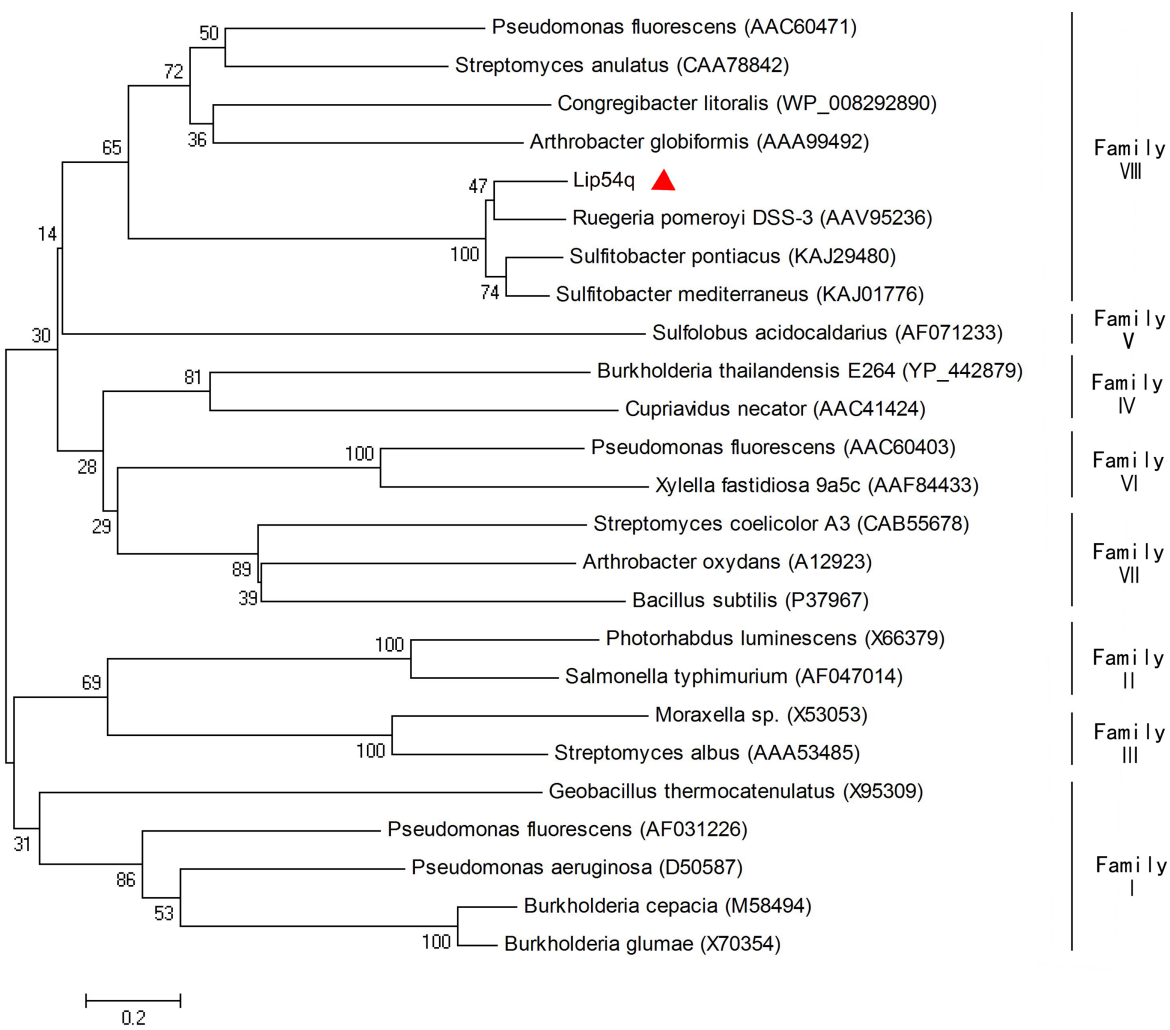
Phylogenetic analysis of Lip54q and closely related lipolytic enzymes. The phylogenetic tree was constructed using MEGA 7.0 software by the neighbor-joining method. All protein sequences were retrieved from NCBI GenBank. The units at the bottom of the tree indicate the number of substitution events. Numbers near a branch show the percentage of reliability in the bootstrap test of that branch.

The positive clone was sequenced, and the sequencing results showed that positive clones contained an insert with a length of 2,480 bp. Using the ORF Finder software on the NCBI website, a new lipolytic enzyme was found. The gene has a length of 1,353 bp and encodes 450 amino acids. The molecular weight of the encoded protein was approximately 48.33 kDa based on theoretical calculations, and the gene was named *lip54q*. A homology analysis of Lip54q with NCBI’s BlastP software showed that the enzyme belongs to the β-lactamase family, which has the highest homology (82%) with a β-lactamase derived from *Donghicola xiamensis*. Clustalx 2.1 and ESPript 3.0 software were used to perform multiple sequence alignments of Lip54q and several proteins with high homology to Lip54q, and the results are shown in Figure 1. Figure 1 reveals that Lip54q contains a conserved S-M-T-K sequence (shown as a black box), which is a typical feature of family VIII in lipolytic enzymes; thus, we inferred that Lip54q belongs to family VIII of lipolytic enzymes. To further confirm our inferences, we performed a phylogenetic analysis of Lip54q and typical members of eight lipolytic enzyme families using MEGA 7.0 software and used the results to construct a phylogenetic tree (shown in Figure 2). The results shown in Figure 2 clearly prove that Lip54q is indeed a member of family VIII of lipolytic enzymes.

### Heterologous expression of *lip54q* and purification of the recombinant enzyme

**Figure 3:**
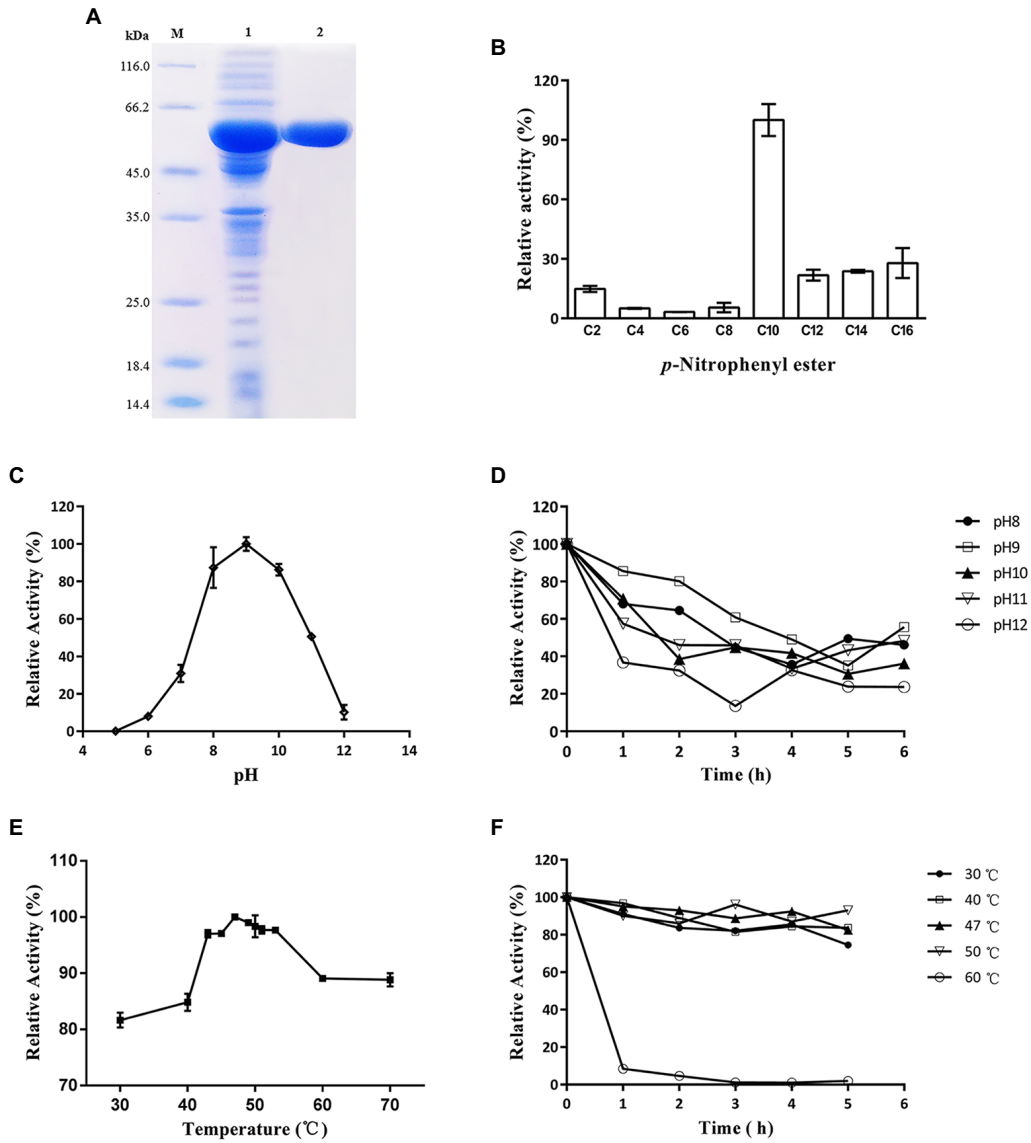
SDS-PAGE analysis and characterization of Lip54q. **(A)** SDS-PAGE analysis of recombinant Lip54q. Lanes: M, standard protein molecular mass markers (size in kilodaltons are indicated on the left;); 1, recombinant Lip54q with his-tag from supernatant of *E. coli* BL21 (DE3) cell lysates; 2, purified recombinant Lip54q with his-tag by affinity chromatography. The target protein band was indicated by arrow. **(B)** Substrate specificity analysis of Lip54q. The enzymatic activity of Lip54q was examined toward *p*-nitrophenol esters with different chain lengths: *p*-nitrophenyl acetate (C2), *p*-nitrophenyl butyrate (C4), *p*-nitrophenyl caproate (C6), *p*-nitrophenyl octanoate (C8), *p*-nitrophenyl decanoate (C10), *p*-nitrophenyl laurate (C12), *p*-nitrophenyl myristate (C14), *p*-nitrophenyl palmitate (C16). The activity of Lip54q toward C10 was regarded as 100%. Data are the average of triplicate experiments, and error bars represent standard deviation. **(C)** Effects of pH on Lip54q activity using *p*-nitrophenyl decanoate as the substrate. Enzymatic activity was measured in 40 mM Britton-Robinson buffer (pH 5.0–12.0) at 45°C. Values are shown as the percentage of maximal activity (100%). **(D)** Effects of pH on Lip54q stability using *p*-nitrophenyl decanoate as the substrate. The residual activity of Lip54q was measured after incubating in 40 mM Britton-Robinson buffer at different pH values (8.0–12.0) for 1–6 h at 45°C. Values are shown as the percentage of maximal activity (100%). **(E)** Effects of temperature on Lip54q using *p*-nitrophenyl decanoate as the substrate. Enzymatic activity was measured at a temperature range from 30 to 70°C in 40 mM Britton-Robinson buffer (pH 7.8). Values are shown as the percentage of maximal activity (100%). **(F)** Effects of temperature on Lip54q stability. The residual activity of Lip54q was measured after incubating in 40 mM Britton-Robinson buffer (pH 7.8) for 1–6 h at various temperatures (30–60°C). Values are shown as the percentage of maximal activity (100%). In Figures 3C–F, the enzymatic activity without incubation was defined as 100%. Data points are the average of triplicate experiments, and error bars represent standard deviation.

The *lip54q* gene was heterologously expressed in *E. coli* BL21 (DE3), and the recombinant enzyme was purified with an Ni-NTA affinity column. After purification, the purity of the crude enzyme solution increased by 9.8 times, and the activity yield was 86.7% (Table 1). To evaluate the molecular weight of the recombinant protein and visually portray the purification effect of the recombinant protein, a crude enzyme solution containing Lip54q and purified enzyme solution were subjected to SDS–PAGE. The results are shown in Figure 3A. Figure 3A shows a high heterologous expression of *lip54q*, and a thick target protein band is shown in the figure. The recombinant protein expression was determined by the Bradford method, and the result was 720 mg/L. In addition, the recombinant enzyme was purified very effectively, and a single band is shown in the figure. The molecular weight of Lip54q was approximately 65 kDa, which was in agreement with the theoretical molecular weight of the recombinant enzyme (the theoretical molecular weight of Lip54q was 48.3 kDa, plus the fusion protein tag on the expression vector of approximately 17 kDa).

**Table 1:** Purification of Lip54q.

Purification step	Total protein (mg)	Total activity (U)	Specific activity (U/mg)	Purification fold	Activity yield (%)
Cell lysate	120.6	21173.7	175.6	1	100%
His-tag affinity chromatography	10.7	18351.7	1715.1	9.8	86.7%

### Characterization of the recombinant Lip54q

The hydrolysis activities of carbon chain substrates with different lengths can be used as a basis to determine whether the lipolytic enzyme is an esterase or lipase. The hydrolytic activities of recombinant Lip54q on carbon chain substrates of different lengths (C2–C16) were tested, and the results are shown in Figure 3B. Figure 3B suggests that the activities of Lip54q on substrates above C10 were significantly higher than those below C10, and the optimal substrate was *p*-nitrophenyl decanoate with a carbon chain length of 10. This indicated that Lip54q is a lipase and not an esterase. Lip54q exhibited the highest activity at pH 9.0 and maintained more than 50% of this highest activity in a pH range of 8.0 to 11.0 but was almost completely inactivated at pH < 7.0 (Figure 3C). These results indicated that Lip54q is an alkaline lipase. Furthermore, when Lip54q was incubated at pH 8.0–11.0 for 6 h, its residual enzyme activity remained more than 40% of the initial enzymatic activity, indicating that the enzyme had good pH stability under alkaline conditions (Figure 3D). The optimum temperature for Lip54q was 47°C, and the activities of Lip54q remained at greater than 80% of the highest activity in the range of 30–70°C, indicating that Lip54q had a wide range of reaction temperatures (Figure 3E). In addition, when the enzyme was incubated for 5 h at or below 50°C, the residual enzyme activities remained greater than 75% of the initial enzyme activity, and the half-life of Lip54q at 50°C was as high as 48 h, which indicated that the enzyme had good thermostability (Figure 3F). The kinetic parameters of Lip54q were measured using the optimum substrate, C10. The results showed that the *V*_max_ and *K*_m_ values of Lip54q were 251.9 μm/min and 0.6 mM, respectively.

### Effects of metal ions, organic solvents, surfactants and other chemical reagents on Lip54q activity

**Figure 4:**
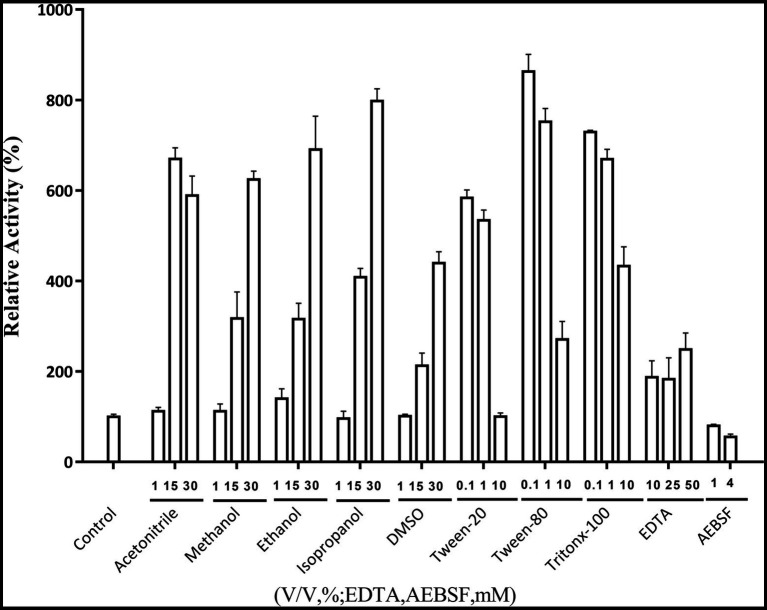
Effects of several organic solvents, surfactants, and two chemical reagents on recombinant Lip54q activities. At the optimal reaction temperature and pH of Lip54q, different final concentrations (10, 25, and 50 mM) of organic solvents (acetonitrile, methanol, ethanol, isopropanol, and DMSO), different final concentrations (0.1, 1, and 10%, v/v) of the surfactants (Tween-20, Tween-80, and Triton X-100), different final concentrations (10, 25, and 50 mM) of EDTA, and two final concentrations (1 and 4 mM) of serine protease inhibitor, AEBSF, were added to the enzymatic reaction system to determine their effects on the enzymatic activities of Lip54q. All tests were repeated three times, and the enzymatic activity of an enzymatic reaction system without the above target reagents was defined as 100%.

The effects of metal ions on Lip54q activity are shown in Table 2. The results in Table 2 show that some low concentrations (1 and 5 mM) of metal ions (such as Ca^2+^, Cu^2+^, Ni^2+^, and Mn^2+^) had strong promotion effects on the enzyme activity of Lip54q, but when these metal ion concentrations increased to 25 mM, their enzyme activities were significantly inhibited. Notably, the enzymatic activity of Lip54q changed only slightly when the Mg^2+^ concentration increased from 1 to 25 mM, indicating that the enzyme was highly tolerant to Mg^2+^, possibly because Mg^2+^ was required for the catalyzed reaction of Lip54q on substrates. In addition, the effects of five organic solvents (acetonitrile, methanol, ethanol, isopropanol, and DMSO), three surfactants (Tween-20, Tween-80, and Triton X-100) and two chemical reagents (EDTA and AEBSF) on Lip54q activities are shown in Figure 4. The results shown in Figure 4 suggest that all five organic solvents strongly promoted Lip54q activity. For example, when the concentrations of DMSO, methanol, ethanol, and isopropanol were increased from 15 to 30%, Lip54q activities increased to 440, 624, 690, and 780% of the initial enzymatic activities, respectively. These results indicated that Lip54q could tolerate high concentrations of organic solvents, and thus, it has very good application prospects in the organic synthesis industry. Low concentrations of the surfactants, Tween-20, Tween-80, and Triton X-100, strongly promoted Lip54 activity; for example, 0.1 and 1% Twewn-80 could increase the activities of Lip54q to approximately 9 and 8 times the initial activity, respectively. However, when the Tween-80 concentration was increased from 1 to 10%, the Lip54q activity was still higher than that of the initial enzyme, but its promoting effect clearly decreased. EDTA is a metal ion chelator and frequently added component in detergents. The results in Figure 4 suggest that EDTA had a certain promoting effect on the enzymatic activity of Lip54q. When the EDTA concentrations were 25 and 50 mM, the enzymatic activities of Lip54q reached 180 and 250% of the initial activity, respectively, indicating that catalytic reactions of Lip54q probably do not require the participation of some divalent metal ions (such as Ca^2+^, Zn^2+^, and Mn^2+^). However, only 4 mM serine protease inhibitor AEBSF significantly inhibited Lip54q activity by 50%. These results revealed that the serine residues in the active catalytic center of Lip54q played a key role in its catalytic function.

**Table 2:** The effects of several different concentrations of metal ions on the activity of recombinant Lip54q.

Metal ions	Concentrations (mM)	Relative activity (%)
Ca^2+^	1	106.9 ± 9.2
	5	132.4 ± 7.4
	25	78.3 ± 2.7
Cu^2+^	1	107.4 ± 5.4
	5	209.0 ± 5.0
	25	11.9 ± 2.9
Ni^2+^	1	122.9 ± 7.9
	5	358.9 ± 9.6
	25	96.9 ± 5.1
Zn^2+^	1	100.8 ± 3.3
	5	260.5 ± 8.5
	25	12.5 ± 1.2
Co^2+^	1	95.8 ± 9.9
	5	347.1 ± 5.1
	25	31.6 ± 8.7
Mn^2+^	1	112.3 ± 6.7
	5	124.8 ± 6.2
	25	16.0 ± 4.4
Mg^2+^	1	101.7 ± 7.5
	5	103.9 ± 3.8
	25	101.5 ± 6.8

### Tolerance of Lip54q to a variety of organic solvents

To determine the tolerance of Lip54q to organic solvents, 12 organic solvents with concentrations of 20 and 50% (log *p* values ranged from −1.35 to 4.7) were used in this study, and the residual Lip54q enzymatic activities were determined after treatments with organic solvents for different times. The results are shown in Table 3. In addition to DMSO and n-butanol, Lip54q enzyme activities increased after 1 h of treatment in 10 organic solvents with 20% concentrations. After 7 days of treatment, residual Lip54q enzymatic activities in organic solvents with low log *p* values (such as DMSO, acetone, methanol, and ethanol) were still 57 to 136% of the original activity. When the organic solvent concentrations were increased from 20 to 50%, hydrophilic organic solvents (log *p* ≤ 0.88) exhibited significant inhibitory effects on enzymatic activities. In addition, Lip54q was extremely stable in organic solvents with 20 and 50% concentrations with high log *p* values, such as chloroform, benzene, toluene, n-hexane, cyclohexane, and n-hexane, and the residual Lip54q enzymatic activity remained greater than 80% of the original activity even after 12 days of treatment. In conclusion, Lip54q is more stable in nonpolar hydrophobic organic solvents than in polar hydrophilic organic solvents (log *p* ≤ −0.24).

**Table 3:** The effects of various organic solvents on the stability of Lip54q.

Organic solvent	Log P	Concentration(%, v/v)	Relative activity (%)			
		1 h	48 h	7 days	12 days
DMSO	−1.35	20	82.5 ± 11.8	118.9 ± 5.4	57.0 ± 4.9	55.3 ± 13.3
		50	37.6 ± 1.4	12.9 ± 1.2	11.9 ± 2.7	10.5 ± 1.6
Methanol	−0.76	20	115.9 ± 9.2	142.4 ± 4.7	136.6 ± 5.7	136.4 ± 7.3
		50	ND	ND	ND	ND
Ethanol	−0.3	20	103.5 ± 7.7	92.9 ± 8.8	91.7 ± 8.1	70.9 ± 1.1
		50	ND	ND	ND	ND
Acetone	−0.24	20	101.4 ± 7.7	98.9 ± 8.0	81.1 ± 6.9	78.0 ± 7.6
		50	39.7 ± 5.1	ND	ND	ND
n-Butanol	0.88	20	32.6 ± 2.0	ND	ND	ND
		50	ND	ND	ND	ND
Chloroform	1.97	20	117.0 ± 5.7	131.2 ± 7.0	86.0 ± 7.5	83.8 ± 7.1
		50	120.4 ± 8.2	115.5 ± 5.2	97.8 ± 10.3	90.5 ± 4.0
Benzene	2.13	20	114.4 ± 6.4	133.4 ± 9.9	92.1 ± 2.8	104.4 ± 1.1
		50	129.8 ± 6.8	136.7 ± 10.3	98.2 ± 5.1	83.2 ± 8.2
Toluene	2.73	20	118.4 ± 17.5	120.8 ± 16.7	88.9 ± 6.7	120.6 ± 12.9
		50	121.0 ± 13.4	124.2 ± 14.4	105.9 ± 3.3	91.1 ± 9.5
Cyclohexane	3.3	20	122.7 ± 15.1	102.9 ± 14.3	87.7 ± 15.7	87.2 ± 9.5
		50	86.6 ± 7.5	99.3 ± 6.7	109.6 ± 7.2	94.1 ± 6.8
n-Hexane	3.5	20	123.0 ± 16.9	127.0 ± 5.8	123.1 ± 5.4	120.3 ± 12.4
		50	144.8 ± 1.6	136.0 ± 4.7	105.0 ± 4.4	84.8 ± 8.4
n-Decanol	4.1	20	127.5 ± 14.5	135.0 ± 8.0	130.2 + 6.8	128.9 ± 3.6
		50	133.4 ± 13.0	104.3 ± 6.2	80.9 ± 21.6	75.5 ± 10.8
Isooctane	4.7	20	131.2 ± 9.2	120.9 ± 6.3	114.2 ± 9.5	100.2 ± 8.6
		50	128.2 ± 12.3	135.4 ± 6.9	98.6 ± 8.4	62.8 ± 4.2

ND, not detected. The log P value is the logarithm of the ratio between the partition coefficients of a substance in n-octane (oil) and water.

### Tolerance of Lip54q to several common commercial detergents

**Figure 5:**
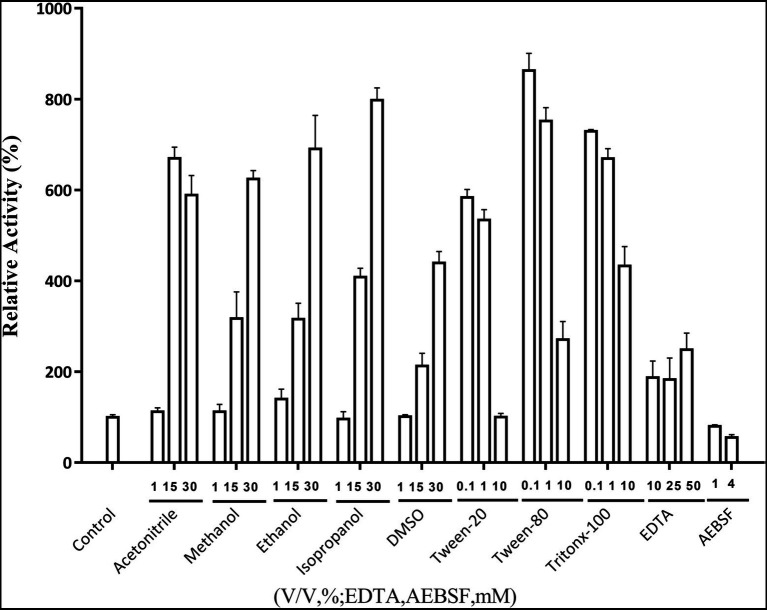
Tolerance of the recombinant Lip54q to six common commercial detergents. Six detergents used in the test include Tide washing powder, Blue Moon laundry detergent, Liby laundry detergent, Walch hand sanitizer, Axe dish soap, and Liby dish soap. The final concentration of Tide washing powder was 10 mg/ml, and the final concentrations of the remaining five detergents were 1% (v/v). Equal amounts (0.5 ml, 10 U) of recombinant enzyme solutions were added to each solution. After 1, 2, 3, 4, 6, and 24 h of treatment at room temperature, the residual activities of each mixture were determined under standard conditions. The enzyme activity of the pure enzyme solution without detergent was defined as 100%, and the relative activities of the enzyme treated with each detergent were calculated. Data points are the average of triplicate experiments.

To explore the application potential of Lip54q as a detergent additive enzyme, six kinds of commercial detergents commonly found in Chinese domestic supermarkets were selected for use in this study. After Lip54q was added to these commercial detergents for certain periods of time, the tolerance of Lip54q to these commercial detergents was investigated by determining the residual enzyme activities. The results are shown in Figure 5. In general, the recombinant enzyme showed good tolerance for different periods to the six detergents, and the residual enzyme activities were still greater than 40% even after 24 h of treatment. In contrast, the recombinant enzyme showed better tolerance to Liby dish soap and Blue Moon laundry detergent, and the residual enzyme activities were still above 70% after 12 h of treatment for these two detergents. Overall, we believe that Lip54q was well tolerated in a wide range of commercial detergents, considering that the enzyme was processed in the detergents for much longer than normal laundry times. Among them, the tolerance of Lip54q to Liby dish soap and Blue Moon laundry detergent was particularly outstanding.

### Immersion resistance of Lip54q to several common commercial detergents

**Figure 6:**
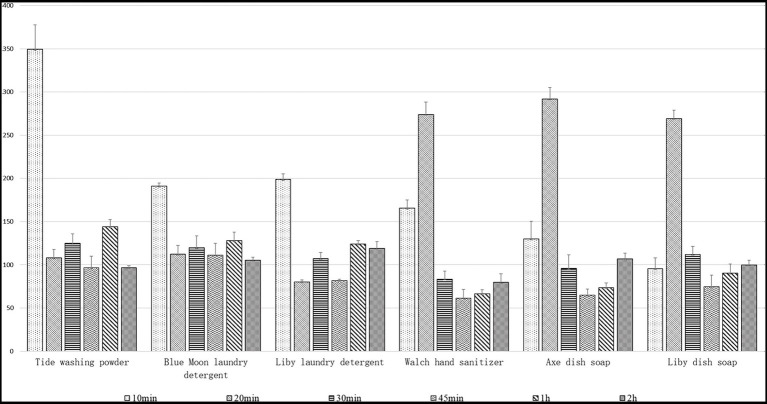
Immersion resistance of the recombinant Lip54q to six common commercial detergents. Six detergents used in the test include Tide washing powder, Blue Moon laundry detergent, Liby laundry detergent, Walch hand sanitizer, Axe dish soap, and Liby dish soap. By using a standard of 10 g of detergent per kilogram of clothing and soaking in 5 l of tap water, six detergents were added to tap water. After mixing, 0.5 ml (10 U) of enzyme solution was added to each experimental group and treated for 10, 20, 30, 45, 60, and 120 min. Then, the residual enzymatic activities were determined under standard conditions. The enzymatic activity of the pure enzyme solution without detergent was defined as 100%, and the relative enzyme activities treated with each detergent were calculated. Data points are the average of triplicate experiments, and error bars represent standard deviation.

In this study, we investigated the immersion resistance of Lip54q to six detergents by adding enzymes and these detergents to the same volume of tap water and then measuring the remaining enzyme activities after soaking for certain periods of time, which simulated daily laundry procedures. The results are shown in Figure 6. In addition to Liby dish soap, the enzymatic activities in the other five detergents after 10 min of immersion had obvious activation effects, and the relative activities of the enzyme were significantly improved compared with those before soaking. However, with an extension of soaking time, the activation effect of each detergent on the enzymatic activities decreased and even had slight inhibition effects on enzymatic activities. Nevertheless, the residual enzyme activities of the six detergent soaking groups after soaking for several different periods of time were still significantly greater than 50% before soaking, indicating that Lip54q has good soaking resistance in a variety of commercial detergents. In addition, optimizing soaking times for different types of detergents could cause Lip54q to have higher residual enzymatic activities, which would result in better laundry performance.

### Oil-stain removal abilities of Lip54q added to several common commercial detergents

**Figure 7:**
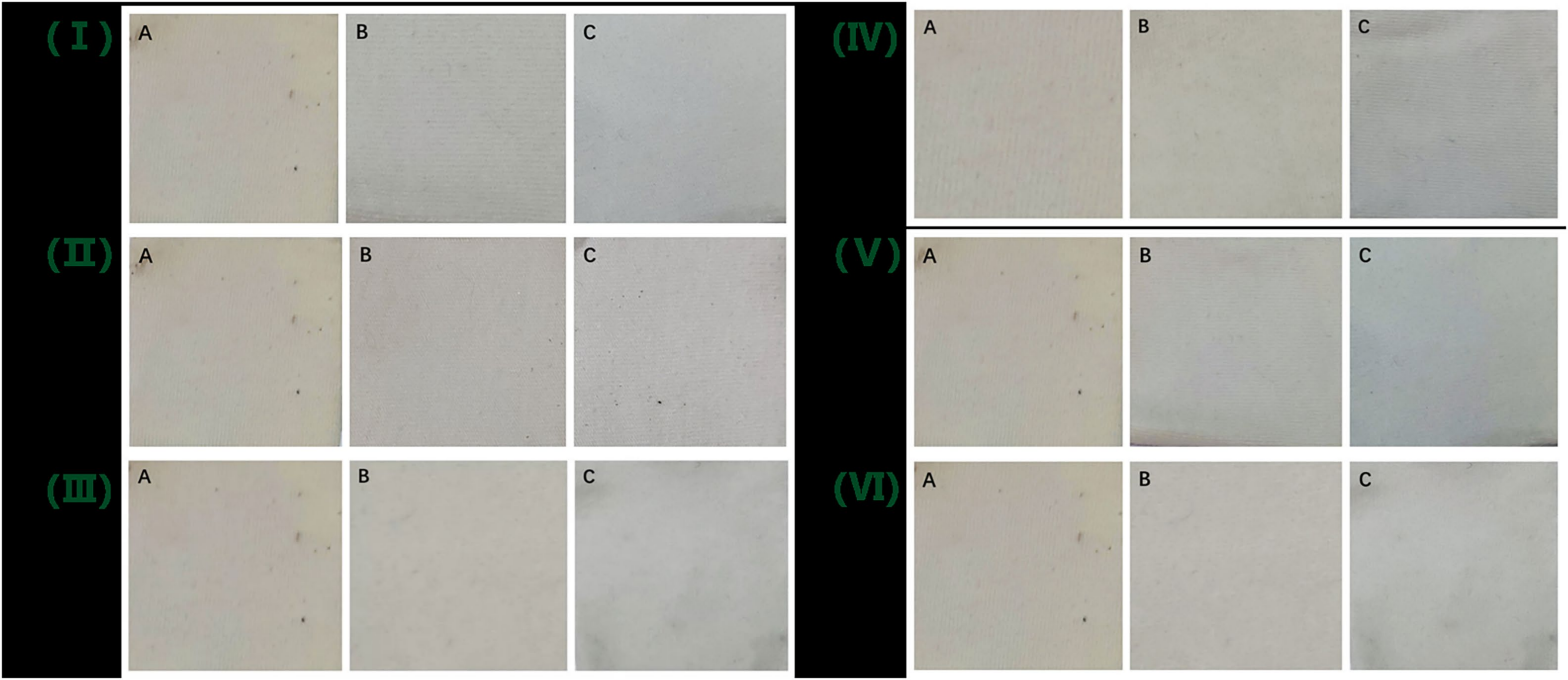
Oil-stain removal abilities of Lip54q added to several common commercial detergents on cotton cloth. Three pieces of white cotton cloth with the same size (4 cm × 4 cm) were soaked in cooking oil for 24 h and then removed to dry naturally. Next, they were numbered as A, B, and C and treated as follows: A. white cloth + tap water, B. white cloth + tap water + detergent, and C. white cloth + tap water + detergent + Lip54q (5 U). The detergents used in this experiment included Tide washing powder (I), Blue Moon laundry detergent (II), Liby laundry detergent (III), Walch hand sanitizer (IV), Axe dish soap (V), and Liby dish soap (VI). The cotton cloths were washed for 30 min using the above treatment methods and then air-dried, and the residual oil stains on the three pieces of white cloth were compared.

To visually demonstrate the oil-removing ability of Lip54q, Lip54q was added to several common commercial detergents to explore its oil-removing ability. The results are shown in Figure 7. Addition of Lip54q to several detergents significantly improved their oil-stain removal abilities. Among them, the oil removal effects were particularly significant in the Liby laundry detergent and Walch hand sanitizer experimental groups. These results demonstrate that Lip54q has good application prospects as a detergent additive enzyme.

### Preliminary analysis of the hydrolysis mechanism of long-chain fatty acids by Lip54q

**Figure 8:**
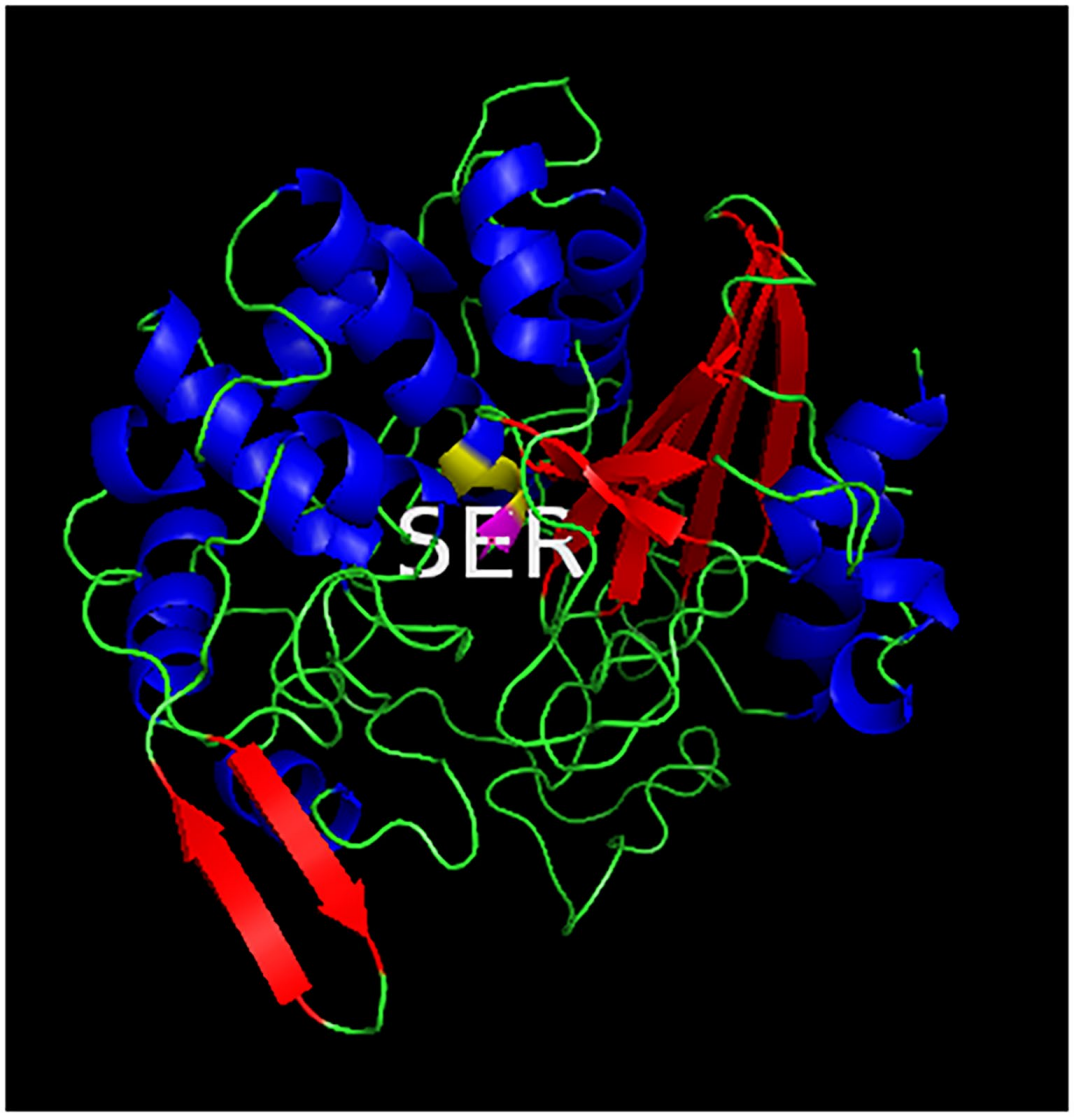
Three-dimensional structure of Lip54q. The homology modeling tool, SWISS-MODEL, was used to construct the 3D structure of Lip54q. The tertiary structure of Est-Y29 with 37.95% homology to Lip54q was use as the template for homology modeling. The blue helix represents the alpha helix structure, the red ribbon represents the β-sheet structure, and the green line represents the random coil. The active center (shown in yellow) of Lip54q contains a Ser residue.

**Figure 9:**
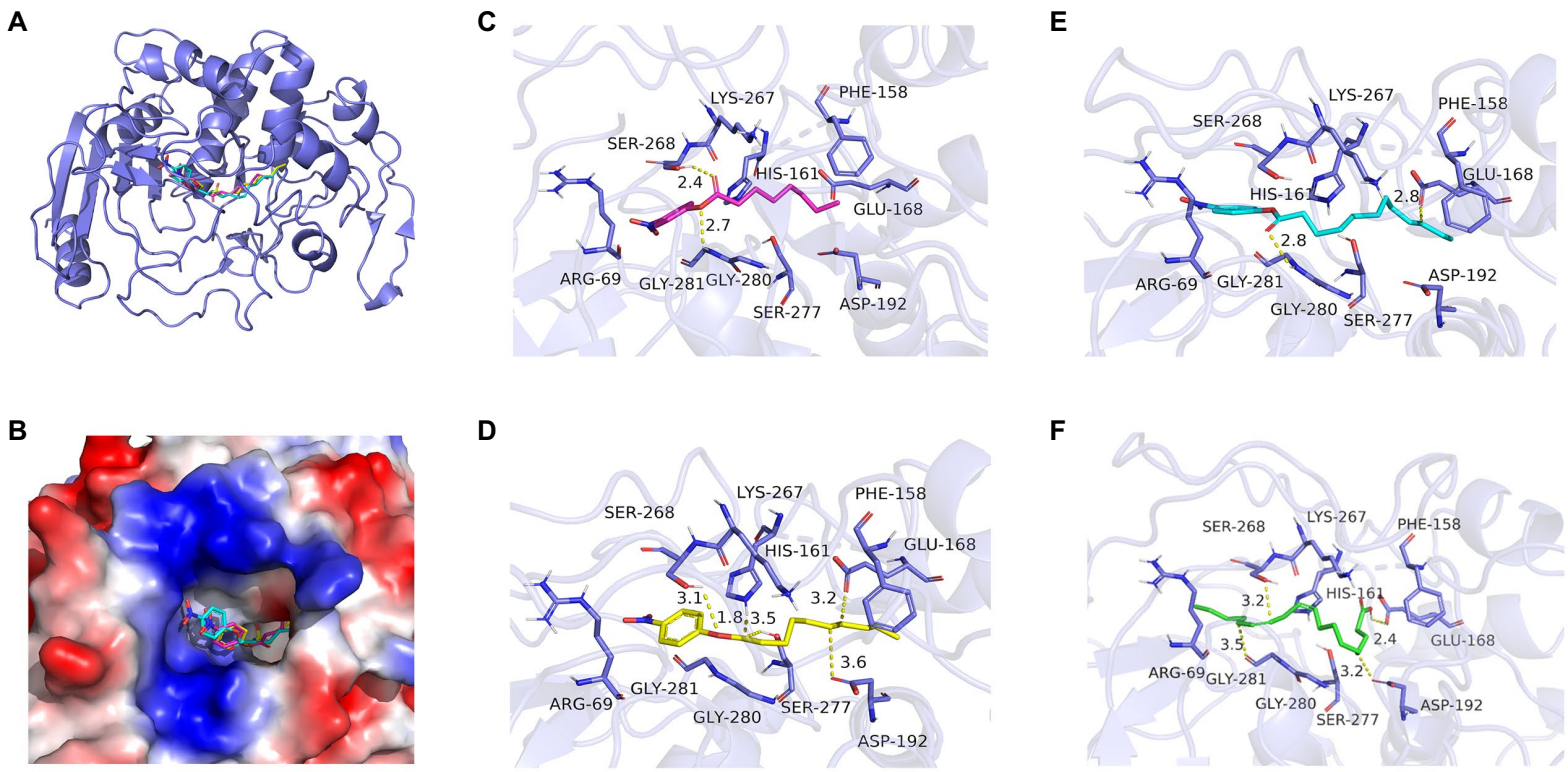
Molecular docking analysis of Lip54q and several substrates. **(A)** The 3D structure of the complex formed by Lip54q and C10 substrate. **(B)** Surface electrostatics of complexes formed by Lip54q and C10 substrates. Red is positive, blue is negative. **(B)** The simulation of electrostatic distributions on the surface of the complex formed by Lip54q and C10 substrate. Blue represents positive charge, and red represents negative charge. **(C)** The intermolecular force analysis of Lip54q bound to C8 substrate. **(D)** The intermolecular force analysis of Lip54q bound to C10 substrate. **(E)** The intermolecular force analysis of Lip54q bound to C12 substrate. **(F)** The intermolecular force analysis of Lip54q bound to linoleic acid substrate. The yellow dashed lines represent hydrogen bonding forces.

Using the online homology modeling software, SWISS-MODEL, the 3D structure of Lip54q was constructed and shown in Figure 8. In the tertiary structures of Lip54q, α-helical structures and random coils accounted for the majority of structures, and there was a distinct beta-sheet structure near the active center. The active center of the enzyme (yellow part) contained a Ser residue, indicating that Lip54q belonged to the typical α/β hydrolase family. To analyze the hydrolysis mechanism of Lip54q for long-chain fatty acids, several substrate molecules (e.g., C8, C10, C12, and linoleic acid) and Lip54q were docked by the Glide module in Schrodinger Maestro software based on the 3D structure of Lip54q. The results are shown in Table 4. The molecular docking results between C8, C10, C12, and linoleic acid substrates and Lip54q showed that the four substrates had good binding interactions with Lip54q with good matching degrees, and the binding energies were less than −5 kcal/mol. The C10 substrate and enzyme had the best performance in terms of binding modes and docking scores, which was consistent with the previous conclusion that C10 is the optimal substrate for Lip54q. The complex formed by the docked substrate molecule and enzyme was visualized by PyMOL 2.1 software, and the binding modes of the substrates and enzyme were obtained (as shown in Figure 9). Amino acid residues binding the substrate molecule to the enzyme pocket could be clearly seen based on the binding patterns. According to Figure 9, the catalytic center site of Lip54q could be composed of three amino acid residues, Ser-268, His-161, and Glu-168 or three amino acid residues, Ser-268, Glu-168, and Asp-192. According to the binding of substrates C8, C10, and C12 to the active pocket of the protein, we found that substrate C10 could form hydrogen bond interactions with Ser-268, His-161, Glu-168, and Asp-192, which could be the catalytic center. The hydrogen bond distances were short, and the binding abilities were strong. Therefore, the C10 substrate in the catalytic center could be effectively anchored; however, C8 and C12 substrates could not form effective interactions with Ser-268, His-161, Glu-168, or Asp-192 in the catalytic center at the same time, which could be the main reason why the enzymatic activity on the C10 substrate was much higher than those on the C12 and C8 substrates. In addition, the results shown in Figure 9 suggest that the linoleic acid molecule matched well with the protein pocket of Lip54q, and the binding region could form hydrogen bond interactions with Ser-268, Glu-168, and Asp-192, which was probably the triplet of the catalytic center, and the hydrogen bond distances were short. This enabled the binding region to form a variety of interactions, such as hydrogen bonds and hydrophobic interactions, which played important roles in the formation of stable complexes between linoleic acid molecules and enzyme proteins, which thus facilitated enzyme protein hydrolysis of linoleic long-chain fatty acids. This could be an important reason for the good oil-stain removal ability of Lip54q.

**Table 4:** The molecular docking results of Lip54q with several long C-chain substrates.

Substrates	Docking score(kcal/mol)	H-bondinteractions	Hydrophobicinteractions
*p*-nitrophenyl octanoate (C8)	−6.2	Ser-268, Gly-281-	Phe-158
*p*-nitrophenyl decanoate (C10)	−7.5	Ser-268, His-161, Glu-168, Asp-192	Phe-158
*p*-nitrophenyl laurate (C12)	−6.2	Gly-281, Glu-168	Phe-158
Linoleic acid	−6.6	Ser-268, Glu-168, Asp-192	Phe-158

## Discussion

Compost is an ideal habitat in which to explore for new lipid hydrolases. In recent years, several lipid hydrolases with unique enzymatic characteristics have been identified from composting habitats (Lu et al., 2019; Park et al., 2020a,b, 2021; Lu and Daniel, 2021). Although these enzymes belong to different ester hydrolase families, including family IV (Park et al., 2020a, 2021; Lu and Daniel, 2021), family V (Lu et al., 2019), and family VIII (Lu et al., 2019; Park et al., 2020b, 2021), the optimal substrates for these enzymes are *p*-nitrophenyl ester substrates with short carbon chains. Therefore, these enzymes are esterases rather than lipases. In this study, we successfully identified a novel lipase gene, *lip54q*, from a metagenomic library of compost soil by using metagenomic technology. Sequence homology analysis indicated that this enzyme belonged to the lipolytic enzyme family VIII. However, different from several lipolytic enzymes previously found in compost, the optimal substrate of Lip54q was C10, and its activities on substrates above C10 were significantly higher than those below C10; thus, Lip54q is a lipase. To the best of our knowledge, this is the first lipase discovered by using metagenomic technology in composting habitats. Since esterases tend to hydrolyze short-chain acyl substrates, whereas lipases hydrolyze long-chain esters and fatty acids (Fojan et al., 2000), Lip54q exhibits a good oil-removing effect compared to several esterases previously found in compost and has good potential as a detergent additive enzyme in the detergent industry.

Homology analysis indicated that Lip54q belonged to the β-lactamase family and shares 38% homology with ESTM-N2, an esterase with β-lactamase activity derived from uncultured microorganisms (Yu et al., 2011). To test whether Lip54q has β-lactamase activity, we examined Lip54q activities using nitrocefin as a substrate. The results showed that although Lip54q belonged to the β-lactamase family, it did not exhibit β-lactamase activity. This result was similar to that reported by Kim et al. for Est2K, an esterase belonging to the β-lactamase family but not having β-lactamase activity (Kim et al., 2010). However, it is different from EstCS3 reported by Park et al., which also belongs to lipolytic enzyme family VIII but has β-lactamase activity (Park et al., 2020b). Studies have shown that whether an esterase of the eighth family of lipolytic enzymes has β-lactamase activity is related to the spatial structure of the esterase itself (Wagner et al., 2002).

Solutions containing detergents are usually highly alkaline (pH 10.0–11.0). Thus, one of the most important enzymatic properties of a lipase suitable for use as a detergent additive enzyme is that it should have good pH stability in highly alkaline environments. In addition, the temperature range for detergent application is generally 30 to 60°C. Therefore, lipases suitable for use as detergent additive enzymes should also have high thermostability in this temperature range. Using metagenomic methods, several lipolytic enzymes with good potential for use in industrial applications have been discovered from compost habitats in recent years. Comparisons of their enzymatic properties with Lip54q are shown in Table 5. The results in Table 5 show that the optimum pH of Lip54q was higher than those for EstCS1 (Park et al., 2020a), Est56 (Lu and Daniel, 2021), Est1 (Lu et al., 2019), and EstCS3 (Park et al., 2020b) and was equal to those for Est2 (Lu et al., 2019) and Est8L (Park et al., 2021), but lower than that of Est13L (Park et al., 2021). In addition, the optimum temperature for Lip54q was similar to those of most esterases but significantly lower than those for Est1 and Est2 (Lu et al., 2019). When comparing the pH stability of Lip54q under strong alkaline conditions (pH 10.0–11.0), Lip54q exceeded all lipolytic enzymes shown in Table 5. When comparing the thermostability of Lip54q in a range of medium and low temperature (30–60°C), Lip54q had lower thermostability than only Est1 and Est2 but significantly exceeded the other lipolytic enzymes. In addition, the substrates required for hydrolysis in the washing industry usually consist of long-chain esters and fatty acids (carbon chain length ≥ 10). Except for Lip54q, the optimal substrates for several lipolytic enzymes shown in Table 5 are short-chain acyl substrates. Considering the above enzymatic characteristics, Lip54q is the most suitable enzyme for use in the washing industry among the lipolytic enzymes found thus far from composting habitats.

**Table 5:** Comparisons of enzymatic properties of Lip54q with several lipolytic enzymes also derived from compost habitats.

Lipolytic enzymes	Optimal pH	pH stability	Optimaltemperature	Thermostability	Optimal substrates	Family of lipolytic enzymes	References
Lip54q	9	pH 8.0–11.0 for 6 h, the residual activity ≥40%	47°C	≤50°C for 5 h, the residual activity >75%	C10	VIII	This study
Est13L	10	ND	40°C	The half-life at 60 ° C was 3.2 min	C6	VIII	Park et al. (2021)
EstCS3	8	Stable within the pH range of 7.0–10.0	55°C	ND	C4	VIII	Park et al. (2020b)
Est2	9	pH = 10, the residual activity = 20%	70°C	50°C for 5 d, the residual activity = 52%	C5	VIII	Lu et al. (2019)
Est8L	9	ND	40°C	The half-life at 50 ° C was 3.2 min	C4	IV	Park et al. (2021)
EstCS1	8	ND	50°C	ND	C3 and C6	IV	Park et al. (2020a)
Est56	8	pH 6.0–8.0 for 24 h,	50°C	50°C for 0.5 h,	C4	IV	Lu and Daniel (2021)
		the residual activity >90%		the residual activity =50%			
Est1	7	pH = 10, the residual activity = 10%	80°C	50°C for 7 d, the residual activity>80%	C4	V	Lu et al. (2019)

ND: no detected.

In addition to stability and thermostability under strong alkaline conditions, as an ideal detergent additive enzyme, it should also have good compatibility with various components, such as surfactants in detergents (Gurkok and Ozdal, 2021). Therefore, in this study, we examined the effects of the nonionic surfactants, Tween-20, Tween-80, and Triton X-100, and the metal ion chelator, EDTA, which is often added to detergents, on the enzymatic activity of Lip54q. The results were compared with those of several lipases with potential applications in the detergent industry. The results are shown in Table 6. These results suggest that 1% (v/v) concentrations of the surfactants, Tween- 20, Tween-80, and Triton X-100, had strong promotion effects on Lip54q activities, and significantly increased the relative activity of Lip54q, and the increases were much greater than those of other lipases (Akmoussi-Toumi et al., 2018; Rmili et al., 2019; Abol-Fotouh et al., 2021; Zhao et al., 2021). When the concentrations of these three surfactants increased to 10% (v/v), Tween-80 and Triton X-100 still significantly increased the relative activity of Lip54q, but Tween-20 did not significantly increase Lip54q activity. The improvement was much greater than those of lipases TA (Abol-Fotouh et al., 2021) and HML (Akmoussi-Toumi et al., 2018). In addition, 50 mM (≈ 1.5%) EDTA significantly promoted Lip54q activity but exhibited inhibitory effects on two other lipases (Akmoussi-Toumi et al., 2018; Zhao et al., 2021). These results suggested that Lip54q has good tolerance to several surfactants and EDTA and has good application prospects in the detergent industry. In addition, we also carried out a tolerance study and soaking resistance study of Lip54q using several common commercial detergents. The results showed that Lip54q had good tolerance to a variety of commercial detergents, and optimized soaking times when using different detergents could further improve the washing effect of the enzyme. These results further demonstrated that Lip54q has good application potential as a detergent additive enzyme.

**Table 6:** Comparisons of tolerance to several surfactants and EDTA of Lip54q with several lipases used for detergent addition.

	Tween 20		Tween 80		Triton X-100		EDTA	References
Lipases	Residual activity (%)	Residual activity (%)	Residual activity (%)	Residual activity (%)	Residual activity (%)	Residual activity (%)	Residual activity (%)
Lip54q	534	101	750	260	669	433	250	This study
	(1% Tween 20)	(10% Tween 20)	(1% Tween 80)	(10% Tween 80)	(1% Triton X-100)	(10% Triton X-100)	(50 mM, 1.5% EDTA)	
TA	112	94	103	84	123	128	92	Abol-Fotouh et al. (2021)
	(5% Tween 20)	(10% Tween 20)	(1% Tween 80)	(10% Tween 80)	(5% Triton X-100)	(10% Triton X-100)	(5% EDTA)	
Lipase from *Bacillus licheniformis*	145.77	ND	123.47	ND	121.57	ND	52.1	Zhao et al. (2021)
NCU CS-5	(1% Tween 20)		(1% Tween 80)		(1% Triton X-100)		(1% EDTA)	
SCL	16 (1% Tween 20)	ND	8 (1% Tween 80)	ND	110 (1% Triton X-100)	ND	ND	Rmili et al. (2019)
HML	ND	101 (10% Tween 20)	ND	105 (10% Tween 80)		102 (10% Triton X-100)	84 (10 mM EDTA)	Akmoussi-Toumi et al. (2018)

ND: Not detected.

In addition to the detergent industry, another important use of lipases is in the organic synthesis industry. Most organic synthesis reactions occur in non-aqueous environments, and thus organic solvent tolerance is a prerequisite for the use of lipases in the organic synthesis industry (Doukyu and Ogino, 2010). In this study, we first examined the effects of five common organic solvents on Lip54q activities. The results showed that low concentrations (1%, V/V) of the five organic solvents had slight activation effects on the activity of this enzyme, and when the concentrations of these organic solvents were increased to 30%, the Lip54q activities were further enhanced. In addition, enzymes are often required to be stored in organic solvents for long periods in industrial applications; thus, we further explored the tolerance of Lip54q to some common polar and nonpolar organic solvents. The results showed that Lip54q was highly stable in 20 and 50% nonpolar organic solvents (log *p* > 0.88), such as chloroform, benzene, toluene, cyclohexane, and n-hexane. The residual activity of Lip54q remained above 80% of the initial activity after being stored in these solvents for 12 days. This result exceeded the performance of other organic solvent-resistant lipases reported in the literature (Jin et al., 2012; Ma et al., 2013). The organic solvent tolerance of Lip54q in 20% polar organic solvents with a low log *p* values (log *p* < 0.88), such as DMSO, acetone, methanol, and ethanol, decreased slightly. However, when the concentrations of these polar organic solvents increased to 50%, they showed strong activity inhibition of Lip54q. For example, the enzyme was inactivated after incubation in 50% methanol or ethanol for 1 h. The stability of Lip54q in hydrophobic organic solvents was greater than that in hydrophilic organic solvents, which is consistent with the characteristics of lipases. Dandavate et al. believed that the tolerance of lipases to organic solvents stems from the natural pressures generated when they hydrolyze water-insoluble long-chain triglycerides; that is, nonpolar organic solvent molecules interact with hydrophobic amino acid residues in the “lid structure” of lipases (the structure covering the catalytic center of the enzyme), thus leaving the conformation of the enzyme in an open state and promoting the catalytic reaction of the enzyme (Dandavate et al., 2009; Ahmed et al., 2010). For example, the lipase, SML, derived from *Stenotrophomonas maltophilia* CGMCC 4254 was very tolerant to nonpolar organic solvents but was very unstable in polar organic solvents (Li et al., 2013).

## Conclusion

In summary, a metagenomic library of compost soil samples was constructed. Through functional screening, a novel lipase, Lip54q, was identified from this library and was heterologously highly expressed in *E. coli*. The recombinant enzyme had good alkali tolerance and temperature tolerance and showed strong tolerance to several surfactants. The results of adding Li54q to several commercial detergents suggested that the enzyme had strong tolerance and soaking resistance to all six detergents. The results indicated that adding the enzyme added to many commercial detergents could significantly improve the oil-stain removal abilities of these detergents. In addition, the results of the molecular docking analysis preliminarily revealed the hydrolysis mechanism of Lip54q for long-chain fatty acid substrates. Due to its good alkaline resistance, excellent compatibility with commercial detergents, and significantly improving the oil removal ability of several commercial detergents, we believe that Lip54q has a good application prospect as an additive enzyme in detergent industry in the future.

## Data availability statement

The datasets presented in this study can be found in online repositories. The names of the repository/repositories and accession number(s) can be found in the article/supplementary material.

## Funding

This work was supported by Science and Technology Program of Guangzhou, China (202201011794); the Guangdong Basic and Applied Basic Research Foundation, China (2020A1515011240); Sun Yat-sen University-Liby transverse subject (71011346); and Sun Yat-sen University-Tidetronbio transverse subject (71011356).

## Author contributions

Q-QL: investigation, methodology, validation, and original draft preparation. Z-RZ: experimental data collection and collation, and software analysis. Q-GL: methodology and investigation. Y-TA: methodology and validation. Y-XW: sample collection and reference query. S-BZ: date curation. GL: project administration, supervision, and review and editing. All authors contributed to the article and approved the submitted version.

## Conflict of interest

Q-GL and S-BZ were employed by company Guang Zhou Liby Enterprise Group Co., Ltd.

The remaining authors declare that the research was conducted in the absence of any commercial or financial relationships that could be construed as a potential conflict of interest.

## Publisher’s note

All claims expressed in this article are solely those of the authors and do not necessarily represent those of their affiliated organizations, or those of the publisher, the editors and the reviewers. Any product that may be evaluated in this article, or claim that may be made by its manufacturer, is not guaranteed or endorsed by the publisher.

## Footnotes

^1^ http://www.ncbi.nlm.nih.gov/

^2^ https://swissmodel.expasy.org/

^3^ https://zhanglab.ccmb.med.umich.edu/I-TASSER/
